# The Relationship Between the Dipping Pattern and Coronary Artery Disease Severity Assessed by the SYNTAX Score in Patients With Hypertension

**DOI:** 10.7759/cureus.36057

**Published:** 2023-03-13

**Authors:** Fatih Kartaler, Mürsel Şahin, Oğuzhan E Turan, Merih Kutlu

**Affiliations:** 1 Cardiology, Soma State Hospital, Manisa, TUR; 2 Cardiology, Medical School, Karadeniz Technical University, Trabzon, TUR; 3 Cardiology, Medical School, Dokuz Eylül University, İzmir, TUR

**Keywords:** non-dipper, syntax score, dipping pattern, coronary artery disease, hypertension

## Abstract

Background

The morbidity and mortality rates related to hypertension (HT) are still high despite the developments in this area. Nondipper hypertension (NDHT) is related to worse clinical outcomes. But the dipping pattern of HT is not still used for treatment targets. In this study, we investigated the effect of dipping patterns on coronary artery disease (CAD) complexity evaluated by the SYNTAX score (SS).

Methodology

Patients with stable CAD and HT were included in the study. All patients were monitored with 24-hour ambulatory monitoring, and dipping patterns were evaluated. Coronary artery complexity was determined by SS for all patients and compared along with different dipping patterns.

Results

A total of 331 patients with HT and stable CAD were evaluated in the study. The mean age of the patients was 62.6 ± 9.9 years, and 172 (52%) were male. The number and percentage of patients with dipper HT (DHT), NDHT, over-dipper HT (ODHT), and reverse-dipper HT (RDHT) were 89 (26%), 143 (43%), 11 (3%), and 88 (26%), respectively. When the groups were compared according to SS, the SS of the patients with RDHT were significantly higher (the SS were 6.33, 4.99, 3.09, and 2.7 for RDHT, ODHT, NDHT, and DHT, respectively, *P* = 0.003). The mean SS between the DHT group and the NDHT group (*P* = 0.03) and between the DHT group and the RDHT group (*P* = 0.01) was significantly different. The less decrease or increase in mean blood pressure (MnBP) values was significantly correlated with high SS.

Conclusions

NDHT, especially the reverse dipping pattern, is closely related to complex CAD. Meticulous consideration of dipping patterns can identify high-risk patients and improve clinical outcomes.

## Introduction

Hypertension (HT) is the most common chronic disease and one of the most important causes of cardiovascular (CV) morbidity and mortality. Despite advances in diagnosis and treatment, deaths and complications related to HT are not well controlled [1]. The most important complication of HT is CV disease. Existing risk factors should be considered when determining treatment targets in patients with HT. Office blood pressure (BP) measurements are generally used in the follow-up of treatment, but it is known that this may be insufficient considering the circadian fluctuations of HT [2]. Therefore, it may be necessary to evaluate ambulatory measurements [3]. It is known that mean BP (MnBP) measurements obtained with 24-hour ambulatory BP monitoring (ABPM) are more closely related to morbidity and mortality [2,4].

A 10% to 20% decrease in BP during sleep is considered physiological and is defined as dipper HT (DHT). A decrease of less than 10% is defined as nondipper HT (NDHT) and has been associated with increased CV mortality. Patients with a decrease in BP of more than 20% during sleep are called over-dipper HT (ODHT), and patients without a decrease but an increase in BP are called reverse-dipper HT (RDHT). Various studies have shown that CV risks are also increased in these patients [5-8].

This study aims to reveal the relationship between the dipping pattern and the severity of CAD in patients with HT. For this purpose, this study was designed to compare the dipping pattern and SYNTAX score (SS); a higher score is indicative of more complex CAD and poor CV prognosis in hypertensive patients [9].

## Materials and methods

This is a cross-sectional study including 331 patients who were admitted to Karadeniz Technical University, Farabi Hospital, between 2015 and 2020 and were diagnosed with CAD and HT. All patients who underwent elective coronary angiography (CAG) and were diagnosed with coronary artery disease (CAD) were performed 24-hour ABPM. Only the patients who had ambulatory BP measurements within 1 month before CAG were included in the study. Again, patients who had ambulatory BP measurements within one month after angiography were included in the study if invasive treatment was not applied. Patients under the age of 18 years; those with heart failure, cardiomyopathy, any rhythm other than sinus, chronic kidney/liver disease, secondary hypertension, sleep apnea, or ongoing pregnancy; and those who were night workers were excluded from the study. Patients with normal coronary arteries or a history of coronary artery bypass surgery were not included in the study. The CAGs were analyzed and evaluated by two interventional cardiologists, and SS were calculated for the lesions ≥1.5 mm diameter and if caused by ≥50% diameter stenosis using the online SYNTAX score calculator 2.10 (www.syntaxscore.com) [10].

Ambulatory BP measurements

The ABPM for 24 hours was recorded by an automatic monitoring device (Mobil-O-Graph NG; I.E.M. GmbH, Stolberg, Germany) and analyzed. Daytime recordings were measured every 15 minutes, and nighttime recordings were measured every 30 minutes. Patients were instructed to press the button before sleeping and after waking up. Otherwise, measurements were obtained as sleep time between 11:00 pm and 08:00 am. Systolic, diastolic, and MnBPs were recorded for both DT and NT. The percentage decrease in BP at night was calculated using the following formula: Nighttime BP reduction (%) = (Mean Daytime BP - Mean Nighttime BP) × 100 / Mean Daytime BP. If the reduction is less than 10%, these individuals were considered to be *nondipper*, and if the difference is 10% or more, these individuals were considered to be *dipper*. SYNTAX scores were also compared among groups with no decrease but an increase in nighttime BP (the reverse dipping group) and those with more than a 20% decrease (the over-dipper group).

Statistical analysis

SPSS (Version 25, IBM Corp., Armonk, NY, USA) was used for analysis. The Kolmogorov-Smirnov test was performed to assess data normality. Continuous variables were presented as mean ± standard deviation (SD) or median with range, and categorical data were expressed as frequencies and percentages by descriptive tests. Analysis of variance (ANOVA), Kruskal-Wallis, Mann-Whitney, and *χ*^2^ tests were used to compare the variables as appropriate. The Bonferroni-corrected Mann-Whitney U test was used for post hoc analysis. The correlation between the SYNTAX score and MnBP reduction percent was analyzed using Spearman's rank correlation coefficient. Univariate and multiple logistic regression analyses were performed to determine the independent predictors (age, hemoglobin A1c [HbA1c], glomerular filtration rate [GFR], interventricular septal thickness [IVS], posterior wall thickness [PW], diabetes mellitus [DM], and SS) of NDHT. The established model was significant with the tests regarding the model fit in the multiple logistics regression analysis (*χ*^2 ^= 8.415; *P *= 0.015). A *P*-value ˂ 0.05 defined the level of statistical significance.

## Results

A total of 331 patients with HT and CAD were evaluated in the study. The mean age of the patients was 62.6 ± 9.9 years, and 172 (52%) were male. DM was present in 92 (27.8%) patients. The number and percentage of patients with DHT, NDHT, ODHT, and RDHT were 89 (26%), 143 (43%), 11(3%), and 88 (26%), respectively. The main characteristics of the groups are shown in Table 1.

**Table 1:** Baseline clinical and laboratory characteristics of patients with HT and CAD. Data are shown as mean ± SD, *n* (%), or median (max-min). ^a^Significance between DHT/RDHT and NDHT/RDHT. ^b^Significance between DHT/NDHT and DHT/RDHT. ^c^Significance between DHT/RDHT. ^d^Significance between DHT/NDHT and DHT/RDHT. DHT, dipper hypertension; NDHT, nondipper hypertension; ODHT, over-dipper hypertension; RDHT, reverse-dipper hypertension; ACEI/ARB, angiotensinogen-converting enzyme/angiotensinogen receptor blocker; GFR, glomerular filtration rate; IVS, interventricular septal thickness; LDL, low-density lipoprotein; LVEF, left ventricle ejection fraction; PW, posterior wall thickness; TIA, transient ischemic attack

	DHT (*n* = 89)	NDHT (*n* = 143)	ODHT (*n* = 11)	RDHT (*n* = 88)	*P*
Clinical characteristics					
Age (years)	60.1 ± 10	63.7 ± 10	63 ± 10	66.1 ± 9	0.021^a^
Female	40 (44.9)	68 (47.6)	3 (27.3)	46 (53.5)	0.357
Diabetes mellitus	12 (13.5)	46 (32.2)	1 (9.1)	33 (37.5)	0.001^b^
Smoker	29 (32.6)	34 (23.8)	3 (27.3)	18 (20.5)	0.289
Stroke/TIA	3 (3.4)	0	0	3 (3.4)	0.158
Dyslipidemia	38 (42.7)	71 (49.7)	5 (45.5)	48 (54.5)	0.465
Heart failure	4 (4.5)	9 (6.3)	0	10 (11.4)	0.227
Laboratory findings					
Total cholesterol (mg/dL)	194.1 (102-321)	193.2 (104-375)	221.8 (97-328)	194.6 (129-267)	0.816
LDL (mg/dL)	116.8 ± 41	115.6 ± 44.7	143.1 ± 50.1	117.7 ± 33.4	0.754
Hemoglobin (g/dL)	13.9 (8-17)	13.8 (11-18)	13.7 (10-18)	13.4 (11-16)	0.374
GFR (mL/min/1.73 m^2^)	86.4 (30-118)	84.5 (32-114)	79.9 (34-113)	81.5 (56-101)	0.0315^c^
Echocardiographic findings					
IVS (mm)	11.8 (9-159)	12.3 (9-18)	12 (8-18)	12.6 (11-14)	0.3377
PW (mm)	10.7 (8-13)	10.9 (8-16)	11.1 (8-18)	12.3 (9-13)	0.193
LVEF (%)	60.5 (30-65)	60.8 (38-65)	64 (30-65)	58.9 (60-65)	0.163
Diastolic dysfunction	74 (86)	116 (82.9)	10 (90.9)	76 (87.4)	0.734
Antihypertensive treatment					
ACEI/ARB	20 (18.9)	45 (20)	45 (20)	0.88	0.801
Diuretic	41 (38.7%)	73 (32.4%)	73 (32.4%)	0.26	0.260
Statin	64 (60.4%)	141 (62.7%)	141 (62.7%)	0.71	0.711
Beta-blocker	64 (60.4%)	139 (61.8%)	139 (61.8%)	0.81	0.818
Calcium channel blocker	39 (36.8%)	80 (35.6%)	80 (35.6%)	0.90	0.907
SYNTAX score	0.88 (0-32)	6.14 (0-36)	6.29 (0-71)	4 (0-25)	<0.0001^d^

The mean age, presence of DM, GFR level, and SS were different between groups. The mean daytime and NT systolic/diastolic BPs of the groups and the percentage reductions in NT systolic and diastolic BPs are shown in Table 2.

**Table 2:** Twenty-four-hour ambulatory blood pressure measurements of the study population. Data are shown as mean ± SD, *n* (%), or median (max-min). ^a^Significance between all groups. ^b^Significance between DHT/NDHT and RDHT/ODHT. MnSBP, mean systolic blood pressure; MnDBP, mean diastolic blood pressure; MnBP, mean blood pressure; SBP, systolic blood pressure; DBP, diastolic blood pressure; DHT, dipper hypertension; NDHT, nondipper hypertension; RDHT, reverse-dipper hypertension; ODHT, over-dipper hypertension

	DHT (*n* = 89)	NDHT (*n* = 143)	ODHT (*n* = 11)	RDHT (*n* = 88)	*P*-value
Daytime MnSBP (mmHg)	125.6 ± 12.5	124.5 ± 14.6	136.3 ± 19	125.6 ± 14.4	0.073
Daytime MnDBP (mmHg)	88.4 ± 9.4	94.7 ± 11.8	84.3 ± 9.4	95.3 ± 12.2	0.088
Daytime MnBP (mmHg)	102.0 ± 10.3	99.8 ± 11.8	100.7 ± 13.5	100.1 ± 11.0	0.454
Nighttime MnSBP (mmHg)	110.9 ± 11.4	119.0 ± 15	105.4 ± 12.2	133.5 ± 15.8	<0.001^a^
Nighttime MnDBP (mmHg)	69.3 ± 8.9	73.7 ± 10.9	66.4 ± 8.1	81.2 ± 10.8	<0.001^a^
Nighttime MnBP (mmHg)	88.4 ± 9.4	94.7 ± 11.8	84.3 ± 9.4	105.3 ± 12.2	<0.001^a^
MnSBP (mmHg)	121.4 ± 12.4	122.8 ± 14.7	129.0 ± 17.6	127.8 ± 14.7	0.013^b^
MnDBP (mmHg)	78.2 ± 9.7	83.4 ± 9.8	83.1 ± 9.4	79.0 ± 9.8	0.850
MnBP (mmHg)	98.1 ± 10.4	98.4 ± 11.8	104.1 ± 12.5	101.5 ± 11.2	0.078
Decrease in SBP (%)	12.1 ± 2.6	4.6 ± 4.3	22.9 ± 5.8	-6.1 ± 5.5	<0.001^a^
Decrease in DBP (%)	15 ± 3.5	6.8 ± 4.2	25.3 ± 4.3	-3.8 ± 5.9	<0.001^a^

**Figure 1:**
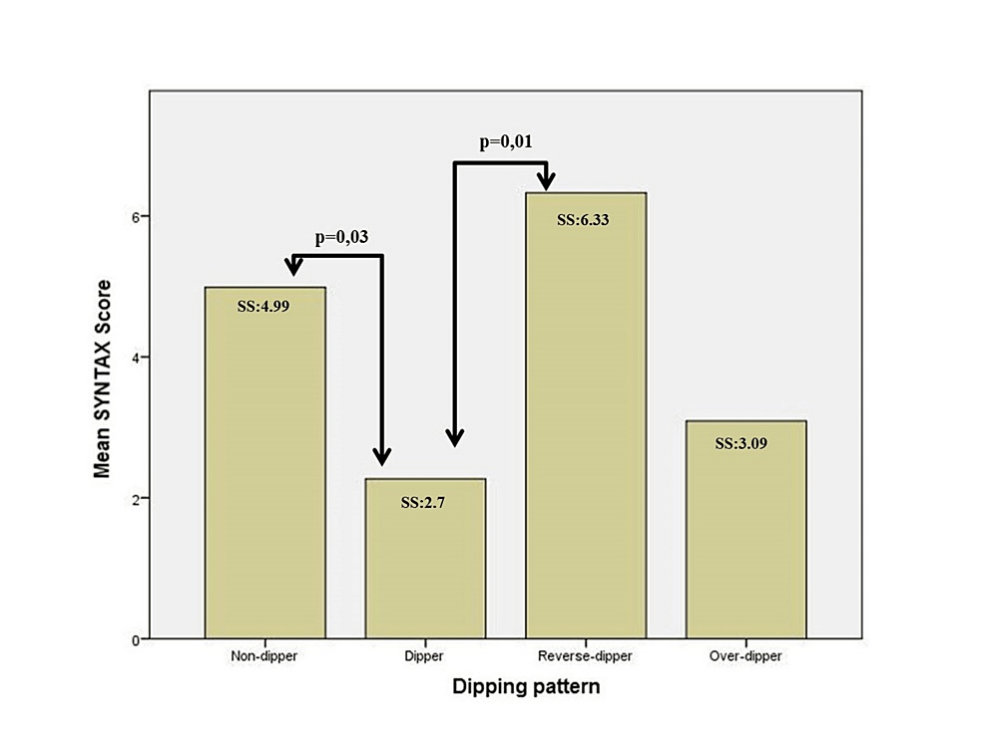
Mean SYNTAX score of dipping pattern groups.

According to MnBPs, all groups could be considered as controlled HT. Daytime mean systolic BP (MnSBP), daytime mean diastolic BP (MnDBP), and daytime MnBP values were similar between the groups, but as expected, nocturnal BP values and percentage reductions were different between groups (*P* < 0.001 for all). When the groups were compared according to SS, the SS of the patients with RDHT were significantly higher (the SS were 6.33, 4.99, 3.09, and 2.7 for RDHT, ODHT, NDHT, and DHT, respectively, *P* = 0.003; Figure 1).

**Figure 2:**
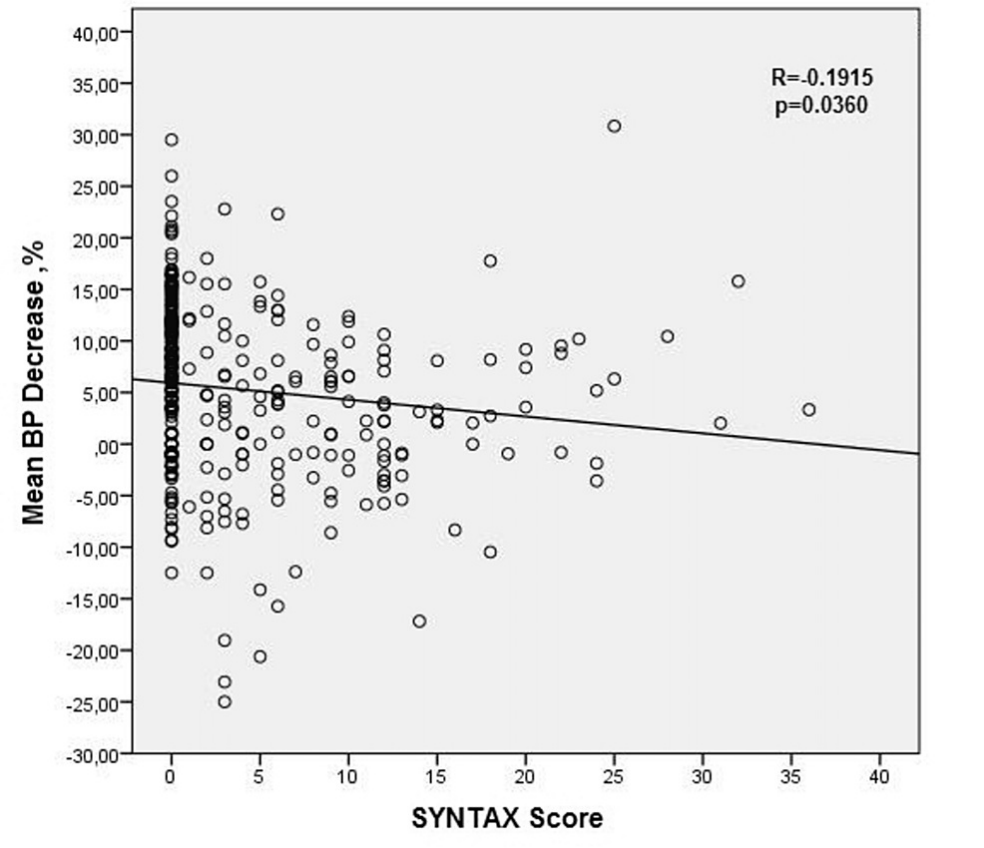
The regression graph of the mean BP reduction and SYNTAX score. BP, blood pressure

The mean SS between the DHT group and the NDHT group (*P* = 0.03) and between the DHT group and the RDHT group (*P* = 0.01) were significantly different. The less decrease or increase in MnBP values was significantly correlated with high SS (Figure 2).

Multivariable analysis showed that NDHT was related to the presence of DM (odds ratio [OR] 5.38, 95% confidence interval [CI] 1.995-14.539; *P* = 0.001), and SS (OR 1.232, 95% CI 1.134-1.338; *P* = 0.007; Table 3).

**Table 3:** Effects of multiple variables on NDHT in multiple logistic regression analyses. DM, diabetes mellitus; GFR, glomerular filtration rate; IVS, interventricular septal thickness; PW, posterior wall thickness; NDHT, nondipper hypertension; CI, confidence interval

	B	Odds ratio	95% CI		P
			Lower	Upper	
Age (Years)	0.020	1.020	0.987	1.054	0.238
GFR	-0.010	0.990	0.971	1.010	0.966
IVS (mm)	0.040	1.041	0.793	1.367	0.772
PW (mm)	0.019	1.019	0.716	1.452	0.915
DM (%)	1.684	5.385	1.995	14.539	0.001
SYNTAX score	0.209	1.232	1.134	1.338	0.0007

## Discussion

The results of this study showed that the SS was significantly different in various dipping patterns in patients with controlled HT. SS was highest in the RDHT group and lowest in the DHT group. The presence of DM and SS were independent predictors for NDHT.

The association between HT and CV risk is well known. The effect of diurnal BP changes on adverse events has also been shown [6,11]. But the dipping pattern has not yet been included in the guidelines for the treatment of HT and risk classification and is not used to determine treatment targets [12-16]. Although many studies are evaluating the relationship between HT and CAD, studies revealing the relationship between circadian fluctuations in BP and the severity of CAD are limited. This was demonstrated in two recently published studies. Mohammed et al. showed that NDHT was an independent predictor of high SS as a continuous variable in 263 acute coronary syndrome patients [17]. The most important controversy of this study was that BP monitoring was done during hospitalization. Harbalıoğlu and Kaypaklı also showed a significant correlation between morning surge and high SS [18]. Our results were similar to both studies with stable CAD and controlled HT patients.

The SS is an important indicator of CAD severity, complexity, and prognosis. The relationship between HT and SS is well known. This study revealed the relationship between dipping status and SS in patients with HT. However, these findings did not reveal a cause-effect relationship. The results suggest that complex CAD may be a cause of adverse clinical events in patients with NDHT and contributed to the need to consider the dipping pattern, not HT alone, in the risk classification of CAD. The findings of this study contributed to the need to consider the dipping pattern, not HT alone, in the risk classification of CAD. Re-evaluation of the dipping pattern after coronary revascularization may reveal a cause-effect relationship. The number of patients in the reverse dipper and over-dipper groups were lower. Data on these patients are also limited in the literature [2,19]. Similar to the literature, the highest SS was observed in the RDHT group, while it was higher in the ODHT group than in the dipper group.

The nondipper rate was found to be 67%. Although this rate was very high, it was similar to previous data in the literature [20-22]. In the most recent comprehensive study, the prevalence of NDHT was found to be 70% in patients with HT under treatment and 75% in patients who were not under treatment [11]. When compared with this study, reverse-dipper rates were higher in our population (35% versus 11%). It seems too high, but it was previously reported between 10% and 40% in several studies among different risk groups such as untreated, diabetic, sleep apnea, and chronic kidney disease patients. A similar prevalence of RDHT (35%) was reported by Afsar et al. among 210 treated HT patients [23]. It is also notable that daytime MnBPs were normal in these patient groups [24].

In previous studies, the presence of DM, old age, decreased kidney functions, and the presence of left ventricular hypertrophy was found to be the independent factors associated with nondipping patterns, and only DM and SS were independent predictors in our study. The exact mechanism of NDHT is not known exactly, but many extrinsic and intrinsic factors are thought to affect it. Physical activity, neurohormonal regulation, nutritional factors, salt consumption, DM, chronic kidney disease, and sleep apnea are some of them [3]. The nondipping pattern in diabetic patients could be associated with renal functions, changes in blood flow, and plasma volume changes caused by hyperglycemia and autonomic dysfunction [11,25]. DM is closely related to complex CAD and NDHT. High SS and the presence of DM were independent predictors of NDHT. Despite their close relationship, this study cannot answer the question if DM and complex CAD is the cause or result of the dipping pattern.

All patients in this study were under treatment for HT. Despite this, the rate of ND pattern was quite high. Previous studies revealed that the ND pattern was seen at similar rates, whether patients are under treatment or not [11]. Although drug administration time differences and the effects of different drug classes are suggested as a reason, conflicting results have been obtained in different studies [26-29]. In this study, no relationship was found between treatment differences and dipping patterns.

Limitations

The findings of this study should be interpreted with limitations. First, it was a single-center, cross-sectional study with a relatively low number of patients, which precluded the generalization of the results. However, the majority of the results were similar to the literature. A single 24-hour ABPM measurement for determining dipping patterns was another limitation. Technical doubts are also important restrictions for 24-hour ABPM measurements. The mean SS was low in the entire population. It would be more appropriate to evaluate the results in a wider SS range.

## Conclusions

Nondipping and reverse dipping patterns are common and closely related to more complex CAD among HT patients. Known prognostic effects on outcomes and the clinical usefulness of dipping patterns in managing HT are limited. This study showed that CAD is more complex among patients with nondipping patterns than dipping ones. Dipping patterns may help determine high-risk patients and optimize treatment goals. The foremost deficiency is that the diagnosis of the dipping pattern is not objective enough. Further studies are needed in this regard.
